# The role of glycoprotein H of equine herpesviruses 1 and 4 (EHV-1 and EHV-4) in cellular host range and integrin binding

**DOI:** 10.1186/1297-9716-43-61

**Published:** 2012-08-21

**Authors:** Walid Azab, Lara Zajic, Nikolaus Osterrieder

**Affiliations:** 1 Institut für Virologie, Freie Universität Berlin, Philippstrasse 13, Haus 18, 10115, Berlin, Germany; 2 Department of Virology, Faculty of Veterinary Medicine, Zagazig University, Zagazig, Egypt

## Abstract

Equine herpesvirus type 1 and 4 (EHV-1 and EHV-4) glycoprotein H (gH) has been hypothesized to play a role in direct fusion of the virus envelope with cellular membranes. To investigate gH’s role in infection, an EHV-1 mutant lacking gH was created and the gH genes were exchanged between EHV-1 and EHV-4 to determine if gH affects cellular entry and/or host range. In addition, a serine-aspartic acid-isoleucine (SDI) integrin-binding motif present in EHV-1 gH was mutated as it was presumed important in cell entry mediated by binding to α4β1 or α4β7 integrins. We here document that gH is essential for EHV-1 replication, plays a role in cell-to-cell spread and significantly affects plaque size and growth kinetics. Moreover, we could show that α4β1 and α4β7 integrins are not essential for viral entry of EHV-1 and EHV-4, and that viral entry is not affected in equine cells when the integrins are inaccessible.

## Introduction

Equine herpesviruses 1 and 4 (EHV-1 and EHV-4) contain a linear, double-stranded DNA genome and belong to the subfamily *Alphaherpesvirinae* in the *Herpesviridae* family. Within the genus *Varicellovirus*, 17 distinct virus species can be identified [1], among them EHV-1 and EHV-4 which are antigenically and genetically closely related [2]. Comparison of the complete DNA sequence of EHV-4 and EHV-1 showed a high degree of similarity that results in a 55 to 96% amino acid identity between proteins of the two viruses. EHV-1 and EHV-4 gH share an amino acid identity of 85% [3]. Despite the similarities with respect to genetic makeup and overall biology, EHV-1 and EHV-4 cause strikingly different pathogenesis beyond the respiratory tract and the observed general malaise following infection.

Initially, EHV-1 infection occurs in epithelial cells of the upper respiratory tract, then the virus spreads to local lymphatic tissues and infects mononuclear cells, which enter the bloodstream and cause productive leukocyte-associated viremia [4-6]. From infected peripheral blood mononuclear cells (PBMC), EHV-1 can initiate viral replication in endothelial cells (EC) lining the small blood vessels of either the nervous system or uterus causing neurological disorders or abortion, respectively [2,7]. EHV-4, on the other hand, generally remains confined to the upper respiratory tract, although the virus has been studied in much less detail than EHV-1. EHV-4 is not commonly associated with leukocyte-associated viremia, infection of PBMC, abortions, or paresis [2,7]. The reduced pathogenic potential of EHV-4 has been attributed to differences in cellular tropism between EHV-1 and EHV-4, since EHV-4 can only infect respiratory epithelial cells and not vascular EC in vivo [7]. Under experimental conditions, EHV-1, but not EHV-4, can infect a broad range of host cells [8]. However, it was recently shown that there was no significant difference in the ability of EHV-1 and EHV-4 to infect equine EC in vitro [7,9].

As with other alphaherpesviruses, EHV-1 entry into cells is presumed to require five viral envelope glycoproteins (gC, gB, gD and the heterodimer gH/gL) and is mediated through different cell surface receptors [9-13]. Depending on the type of cell infected, entry of EHV-1 can occur via endocytosis or fusion at the plasma membrane [6,14,15]. Among herpesviral glycoproteins gB, gH, and gL are conserved across all herpesviruses and, consequently, essential for virus entry and cell fusion. Several studies suggested that gH/gL itself has fusogenic properties [16,17]; however, the recently determined crystal structures of herpes simplex virus type 2 (HSV-2) gH/gL, Epestein-Barr virus (EBV) gH/gL, and pseudorabies virus (PRV) gH revealed that gH/gL does not resemble any known fusion protein. Instead, it may act as a fusion regulator [18-21]. The structural studies showed that gH has three distinct domains with the N-terminal domain (domain H1) shown to bind to gL [20]. It has long been known that, in the case of EBV and HSV, gL is required for correct folding, trafficking, and function of gH [19,22]. How gB and gH/gL function and interact during alphaherpesviral fusion is still not fully understood. Recent studies suggest that fusion is a stepwise process starting with gD binding to its cognate receptors, followed by activation of gH/gL to prime gB for fusion [23,24].

Integrins are heterodimeric cell adhesion receptors that mediate cellular interactions with the extracellular matrix and surrounding cells, which serve as receptors for many viruses [25,26]. An integrin-binding motif present in gD of EHV-1, arginine-serine-aspartic acid (RSD), was shown to facilitate endocytosis through interactions with αVβ5 in CHO-K1 or αVβ3 in PBMC. However, the motif present in gD of EHV-4, RSN, does not lead to significant PBMC infection [6]. That being said, even when this motif was mutated in EHV-1, infection of PBMCs was never completely abolished, meaning that endocytosis and infection of PBMCs is a complex, multistep process, which may require other viral glycoproteins and cell surface molecules.

One of these glycoproteins may be gH, which harbors a motif similar to that shown to mediate binding of rotaviruses to α4 integrins [27,28]. The amino acid motif leucine-aspartate-valine (LDV) is the primary binding site for α4β1 integrins in fibronectin, and the related motif, leucine-aspartate-isoleucine (LDI), has been shown to bind α4 integrins as well [27,28]. The most remarkable feature of α4 integrin-binding sites is the presence of an aspartate residue next to a hydrophobic residue on the amino- or carboxy-terminal side of aspartate [29]. Therefore the serine-aspartate-isoleucine (SDI) sequence found at amino acid residues 457–459 in EHV-1 possibly represents an α4 binding motif. EHV-4, on the other hand, has a single nucleotide substitution at position 447 relative to EHV-1, which changes this motif to alanine-aspartate-isoleucine (ADI). Interestingly, α4 integrins are dominantly and highly expressed on B and T lymphocytes as well as on monocytes [30].

We here investigated the role of EHV-1 and EHV-4 gH in infection, cellular entry and/or host range and integrin binding. To this end, the gH genes were exchanged between EHV-1 and EHV-4 via two-step Red-mediated recombination of infectious clones of either virus [31]. In addition, the putative SDI integrin-binding motif within EHV-1gH was mutated as it was hypothesized to be important in cell entry through binding to α4β1 or α4β7 integrins.

## Materials and methods

### Viruses

EHV-1 strain L11Δgp2 and recombinant WA79 derived from an infectious EHV-4 BAC clone were generated and used as described before [32,33].

### Plasmids

Transfer plasmids encoding either EHV-1 or EHV-4 gH with a kanamycin-resistance (*kan*^*R*^) gene were constructed. All primers used are listed in Table 1. EHV-1 or EHV-4 gH genes were amplified by polymerase chain reaction (PCR) using primers P1, P2, P3, and P4 (Table 1), respectively. PCR products were digested with restriction enzymes and inserted into the pcDNA3 vector (Invitrogen, Darmstadt, Germany), resulting in recombinant plasmids pcDNAgH1 and pcDNAgH4. To construct pcDNAgH1-Kan and 4-Kan, the *kan*^*R*^ gene was amplified from the pEPkan-S plasmid [31] using primers P5, P6, P7 and P8 (Table 1). PCR products were digested and inserted into pcDNAgH1 or pcDNAgH4. Correct amplification and insertion were confirmed by nucleotide sequencing (Starseq, Mainz, Germany).

**Table 1:** Olignucleotide primers used in this study

**Primer**	**Product**	**Sequence**
P1	EHV-1gH	*tat ***ggatcc**atgttacaaccgtatcgaaa^a^
P2		*ata***gggccc**ttactcataactcaataaca^a^
P3	EHV-4gH	*tat***ggatcc**atgtcacaaccgtatctaaa^a^
P4		*ata***gggccc**ttactcagagtttaataaca^a^
P5	Kan-1	ggc**gcggccgc**cggttcgcatgttatcctcaaggatgacgacgataagtaggg^a,b^
P6		ccg**gcggccgc**gccaacagcaaaaaatagcacaaccaattaaccaattctgattag^a,b^
P7	Kan-4	tat**tctaga**aagccgctaccaaacgcggtaggatgacgacgataagtaggg^a,b^
P8		ctt**tctaga**ataagcgtacacgcttttcacaaccaattaaccaattctgattag^a,b^
P9	gH1-deletion	gtggctgtacattaacttgggaatcattacttccgcgatcacaaatatcccgtgtgttgtaggatgacgacgataagtaggg^b^
P10		attgtctaacatggggggtaacaacacacgggatatttgtgatcgcggaagtaatgattccaaccaattaaccaattctgattag^b^
P11	gH4-deletion	atccagtggttgtatattgggaataaatactgctgcgattacacaaacaatgtctagtgtaggatgacgacgataagtaggg^b^
P12		ctgtttacacgcaatacaacacactagacattgtttgtgtaatcgcagcagtatttattccaaccaattaaccaattctgattag^b^
P13	gH4-Kan	aagatataccgtggctgtacattaacttgggaatcattacttccgcgatcatgtcacaaccgtatctaaa
P14		tcgcacaaatattgtctaacatggggggtaacaacacacgggatatttgtttactcagagtttaataaca
P15	gH1-Kan	tactcgaggtatccagtggttgtatattgggaataaatactgctgcgattatgttacaaccgtatcgaaa
P16		actcgtatactgtttacacgcaatacaacacactagacattgtttgtgtttactcataactcaataaca
P17	gH^440A^	ccagtttgacgttgcacaatcccagattgagaaaatagtg**G**cagatatcaacgtggaggccaggatgacgacgataagtaggg^b,c^
P18		tacatcggtttgcgcaattcggcctccacgttgatatctg**C**cactattttctcaatctgggcaaccaattaaccaattctgattag^b,c^
P19	Primers for sequencing	tgtgacctggattcatttag
P20		aacaattttacgcgtaatat
P21		ttgtgctcttaaatcattta
P22		gccgcattcccgtttatagc
P23		taatgtcaaaaaatctttta
P24		gtttacgtactcagcgatgg
P25		ccatacgtgatataactgat
P26		gtgaggataacatgcgaacc
P27		atgacaatttggagccgttt
P28		tatgcaggacctatctacaa
P29		actataggctttgctatatt

^a^Restriction enzyme sites are given in lower case bold letters; sequences in italics indicate additional bases which are not present in the EHV-1 or -4 sequence.

^b^Underlined sequences indicate the template binding region of the primers for PCR amplification with pEPkan-S.

^c^Upper case bold letters indicate the nucleotides that were mutated.

### Cells

Fetal horse kidney (FHK) cells, kindly provided by Dr V. Svansson, University of Iceland, human embryonic kidney (293), RK13, and Vero cells were propagated in Dulbecco’s modified Eagle’s medium (DMEM, Biochrom, Berlin, Germany) supplemented with 10% fetal bovine serum (FBS, Biochrom). Equine dermal (NBL-6) and CHO-K1 cells were grown in Iscove’s modified Dulbecco’s medium (IMDM, Invitrogen) supplemented with 10% FBS. CHO-A, CHO-B, and CHO-C were generously provided by Dr P. Spear, Northwestern University, Chicago, IL, and express the HVEM, nectin-2 and nectin-1 receptors, respectively. They were grown in IMDM supplemented with 10% FBS and 500 μg/mL G418 (Invitrogen). PBMC were isolated from heparinized blood collected from healthy horses as described before [9]. After two washing steps, cells were resuspended in RPMI 1640 (Biochrom) supplemented with 10% FBS. For generation of RK13 cells, which constitutively express EHV-1 gH (RK/gH1), RK13 cells were transfected with the recombinant pcDNAgH1 plasmid using Lipofectamine 2000 (Invitrogen) and colonies resistant to G418 (800 μg/mL) were selected and analyzed by western blotting for expression of EHV-1 gH. As controls, cells were transfected with the pcDNA3 vector (RK/vector) and were also maintained in medium containing G418.

### Antibodies

The anti-mouse DATK32 monoclonal antibody (MAb), an α4β7 integrin antagonist, and MAb P4C2, an α4β1 integrin antagonist, were obtained from Biolegend (Fell, Germany) and Abcam (Cambridge, UK), respectively. Isotype-matched mouse immunoglobulin G (IgG) was used as a control (Cell Signaling Technologies, Frankfurt am Main, Germany). EHV-1 polyclonal anti-gH antibodies were kindly provided by Dr Antonie Neubauer-Juric, Bavarian Health and Food Safety Authority, Germany.

### BAC Mutagenesis

EHV-1 strain RacL11 cloned as a BAC (pL11) [32] and EHV-4 BAC clone pYO03 [33] were described earlier. pL11 and pYO03 BACs were maintained in *Escherichia coli* (*E. coli*) GS1783 cells (a kind gift from Dr Greg Smith, Northwestern University, Chicago, IL, USA). Deletion of gH1 and gH4 was done via two-step Red recombination as previously described [31]. Briefly, PCR primers, P9, P10, P11, and P12 (Table 1), were selected such that the 50 nucleotide (nt) recombination arms enabled the substitution of the gH gene by the *kan*^*R*^ gene at *nt* 1 to 2546 or 1 to 2567 in EHV-1 or EHV-4, respectively. PCR products were digested with *Dpn*I in order to remove residual template DNA. Transfer fragments were then electroporated into GS1783 containing the BACs. Kanamycin-resistant colonies were purified and screened by PCR and RFLP to detect *E. coli* harboring mutant clones. Positive clones were subjected to a second round of Red recombination to obtain the final constructs, pL11ΔgH1 and pYO∆gH4, after excision of the *kan*^*R*^ gene.

The transfer constructs gH4Kan and gH1Kan were amplified by PCR using pcDNAgH4Kan or pcDNAgH1Kan templates and primers P13, P14, P15, and P16 (Table 1). PCR products were electroporated into GS1783 harboring pL11 or pYO03. After selection on LB agar plates containing 25 μg/mL chloramphenicol and kanamycin, resistant colonies were purified and screened by PCR and RFLP to detect *E. coli* harboring recombinant pL11gH4Kan and pYOgH1Kan. Positive clones were subjected to a second round of Red recombination to obtain the final constructs pL11gH4 and pYOgH1.

A point mutation targeting the SDI motif present in EHV-1 gH was engineered by converting nucleotide 1318 of gH from a thymidine to a guanine, changing the serine into alanine (gH^S440A^), by employing two-step Red-mediated recombination. Primers, P17 and P18, used for mutant generation are listed in Table 1. The respective genotypes of all the mutants and revertants were confirmed by PCR, RFLP, and nucleotide sequencing using primers P19-P29 (Table 1).

### Generation of recombinant viruses

EHV-1gH4 and EHV-1gH^S440A^ were reconstituted after transfection of purified BAC DNA into RK13 cells [32]. For EHV-4gH1, the virus was reconstituted by transfection of purified DNA into 293 cells as described earlier [33,34]. After three days, the supernatant and cells were collected and used to infect confluent NBL-6 cells.

### Western blot analysis

For western blot analyses, pellets of infected FHK cells or RK/gH1 were resuspended in radioimmunoprecipitation assay buffer with a protease inhibitor cocktail (Roche, Mannheim, Germany). Sample buffer (1M Tris/HCl, pH 6.8; 0.8% sodium dodecyl sulfate (SDS); 0.4% glycerol; 0.15% β-mercaptoethanol; 0.004% bromophenol blue) was added, the mixture was heated at 95°C for 5 min and proteins were separated by 10% SDS-polyacrylamide gel electrophoresis (PAGE) exactly as previously described [35]. Expression of gH was detected using EHV-1 polyclonal anti-gH antibodies and goat anti-rabbit IgG coupled to peroxidase (Southern Biotech, Eching, Germany) as secondary antibodies. A Rabbit anti-β-actin antibody (Cell signaling Technologies) was included as a loading control. Reactive bands were visualized by enhanced chemoluminescence (ECL plus, Amersham, Freiburg, Germany).

### Virus growth assays

To determine viral replication, single-step growth kinetics and plaque diameter assays were performed as described before [35,36]. Briefly, confluent NBL-6 cells were infected at a multiplicity of infection (MOI) of 1 or 0.01 for 1 h at 37°C. Cells were then washed and overlaid with DMEM with 10% FBS. Infected cultures were harvested at the indicated times post-infection (pi) and viral titers were determined by plating onto NBL-6 cells. Plaques were counted and growth kinetics were determined in three independent experiments. For plaque size measurements, NBL-6 cells were infected with viruses (MOI of 0.001) and overlaid at 1 h pi with DMEM containing 0.5% methylcellulose (Sigma, Hamburg, Germany). At 3 days pi, 50 fluorescent plaques were photographed for each virus and average plaque diameters were calculated using ImageJ software vl.32j [37]. Values were compared to plaque areas induced by parental viruses, which were set to 100%. Three independent experiments were used to calculate average plaque sizes and standard deviations.

### Virus infection assay

For measuring infectivity (efficiency of plating) of recombinant viruses, cell monolayers were inoculated with different viruses (MOI 0.1). After 1 h of adsorption, cells were washed and overlaid with DMEM containing 10% FBS and infection was allowed to proceed for 48 h. Cells infected with each virus were monitored and photographed with an Axiocam CCD camera (Zeiss, Berlin, Germany).

### Flow cytometry

To evaluate integrin expression, NBL-6, FHK, and PBMC cells were incubated with 2 μg/mL of the anti-integrin MAbs targeting α4β7 (DATK32) or α4β1 (P4C2) or an isotype control mouse IgG for 1 h at RT. After washing twice with PBS, cells were incubated with Alexa fluor 488-labeled goat anti-mouse IgG (1/500 dilution) for 1 h at RT. After a final wash, 10 000 cells were analyzed using a FACScalibur flow cytometer (BD Biosciences, Heidelberg, Germany), and the intensity of fluorescence was analyzed using FlowJo software (Treestar, Olten, Switzerland).

For infection experiments, 2 × 10^5^ cells were prepared in 24 well plates as monolayers (NBL-6 and FHK) or as suspensions (PBMCs). Before adding the antibodies, media was removed and cells were washed with PBS containing 2% FBS. Cells were incubated with integrin antibodies (20 μg/mL) at 4°C for 1 h [6,9]. Cells were washed and infected with viruses at an MOI of 2 or 5. NBL-6 and FHK cells were trypsinized at 24 h pi and washed twice, while PBMC were washed twice at 48 h pi. After centrifugation, cells were resuspended in PBS containing 10 μg/mL propidium iodide (PI; Invetrogen) and the intensity of fluorescence of 10 000 cells was analyzed to determine the percentage of infected cells.

### Statistical analysis

Using Microsoft Excel, Student’s *t* test for paired data was used to test for significance. Data given are as mean values, and bars show standard deviations.

## Results

### Generation and genotypic characterization of mutant viruses

**Figure 1:**
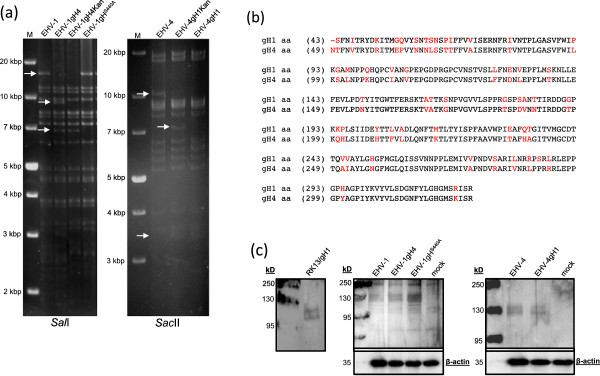
**Identification of recombinant viruses by RFLP and Western blotting.** (**a**) Purified DNA from EHV-1, EHV-1gH4, EHV-1gH4Kan and EHV-1gH^S440A^ (left panel) as well as EHV-4, EHV-4gH1Kan and EHV-4gH1 (right panel) were digested with *Sal*I and *Sac*II. Fragments in the mutants that appeared as a consequence of the deletion or insertion of gH sequence are marked by arrows. (**b**) Amino acid sequence alignment of EHV-1 and EHV-4 gHs’ N-terminal part. The alignment begins with aa49 to aa 327 (H1 domain homologue, that binds gL, in HSV-2) [20]. (**c**) Parental and mutant viruses as well as complementing RK13/gH1 cells express gH at similar levels. Cell lysates were prepared either from infected FHK cells or from RK13/gH1 and proteins were separated by SDS-10%-PAGE. The blots were incubated with EHV-1 polyclonal anti-gH antibody and detected with anti-rabbit IgG peroxidase conjugate. For RK13/gH1 cells (left panel), EHV-1 and related mutants (middle panel), and EHV-4 and related mutants (right panel), two bands of approximately 125 and 115kD were detected that are not present in mock-infected cells. β-actin was used as a loading control.

Two-step Red-mediated recombination was used to exchange the gH-encoding sequences between EHV-1 and EHV-4. The newly generated viruses included an EHV-1 mutant harboring (gH4) in place of gH1, and a corresponding EHV-4 carrying gH1. Furthermore, EHV-1 with a point mutation in the SDI motif present in gH was engineered by changing the serine into alanine (gH^S440A^). PCR analysis showed that all constructs harbored the desired genetic modifications (data not shown) and further analysis of the mutant clones was done using nucleotide sequencing (data not shown). gH1 is located within a 15.1 kbp *Sal*I fragment, which disappeared and was replaced by two bands of 8.3 kbp and 6.8 kbp in size in the case of EHV-1gH4 due to the presence of a *Sal*I site in the gH4 gene. The presence of the *kan*^*R*^ gene, in EHV-1gH4Kan added 1 kbp to the larger band, and a fragment of around 9.3 kbp in size, which is difficult to identify as it comigrated with another, slightly larger fragment (Figure 1a). On the other hand, the gH4 gene is located in a 10-kbp *Sac*II fragment. Replacement of gH4 with gH1 resulted in two bands of 6.2 kbp and 3.5 kbp is size as the gH1 sequence contains two *Sac*II sites. The 6.2 kbp comigrates with several bands around 6 kbp in the *Sac*II restriction digest of EHV-4. However, the presence of *kan*^*R*^ gene resulted in a higher band of around 7.2 kbp in size in the EHV-4gH1Kan (Figure 1a).

To determine whether gH1 and gH4 were expressed properly by the mutant viruses, FHK cells were infected with the respective viruses and infected-cell lysates were analyzed by western blotting. Our results showed that gH, approximately 125-kD in size, was expressed by all viruses including the mutants (Figure 1c).

### Virus growth analyses

**Figure 2:**
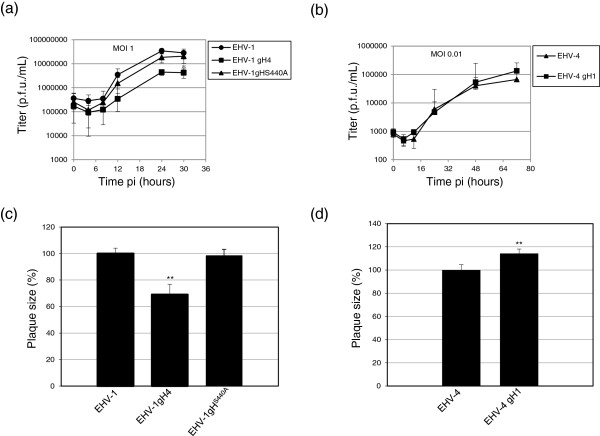
**In vitro growth characterization of parental and mutant viruses.** (**a**, **b**) For growth kinetics, NBL-6 cells were infected at MOI’s of 1 or 0.01. Infected cells and supernatants were collected and virus titers were determined at the indicated times pi. The data presented are means ± SD of triplicate measurements. The asterisk indicates a *P* < 0.05 for means when compared to the parental viruses. (**c**, **d**) Means ± SD of diameters of 50 plaques measured for each virus are shown. The plaque diameter of parental viruses was set to 100%. The asterisks indicate a *P* < 0.05 for means when compared to the parental viruses.

Three independent growth kinetic experiments and plaque size measurements were performed using equine NBL-6 cells. Compared to parental EHV-1, EHV-1gH4 had a significantly reduced growth rate over the 30 h time course (Figure 2a). In contrast, the growth rate of EHV-1gH^S440A^ was not significantly different from that of the parental virus control (Figure 2a). With regard to the EHV-4-based recombinant, both viruses grew to almost identical titers in NBL-6 cells (Figure 2b). In addition, the EHV-1 mutant harboring gH4 produced plaques significantly smaller in size when compared to plaques formed by EHV-1 parental virus and reached only 69% of the plaque diameters relative to those induced by EHV-1 (Figure 2c). However, plaques formed by EHV-1gH^S440A^, on average, reached 98.4% of the size of parental virus plaques, a reduction that was not significant relative to parental virus. We concluded from the experiments that there was no significant difference in plaque size due to the mutation of the putative integrin-binding motif SDI (Figure 2c). In contrast, insertion of gH1 in place of the authentic glycoprotein gH4 in EHV-4 led to an increase in the average plaque diameter of the mutant virus, which was 114.3% relative to that of EHV-4 parental virus (Figure 2d), indicating that gH may also have a role in cell-to-cell spread.

### EHV-1 gH is essential for virus replication

**Figure 3:**
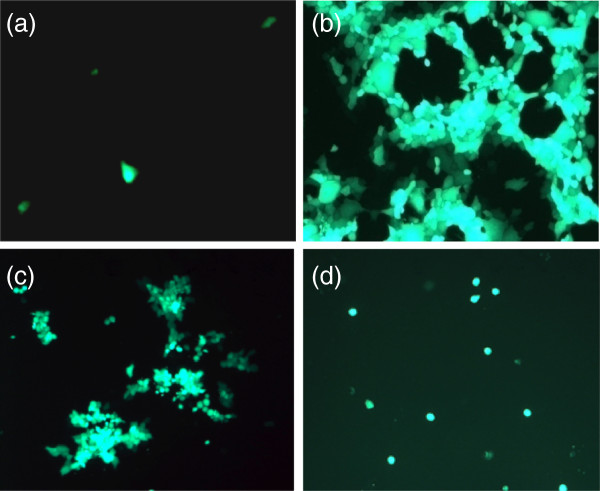
**Infection of RK13 cells with EHV-1ΔgH.** RK13 cells were transfected with either EHV-1ΔgH DNA (**a**) or EHV-1 parental DNA (**b**). EHV-1ΔgH was also transfected into RK13/gH1 cells (**c**). Recovered virus was collected from RK13/gH1 cells and used to infect naïve RK13 cells (**d**). Infected cells appear green as all viruses express EGFP. Cells were inspected with a fluorescent microscope (Zeiss Axiovert) and images were taken with a CCD camera (Zeiss Axiocam). The bar represents 100 μm.

When DNA of EHV-1ΔgH was transfected into RK13, virus replication remained restricted to a small number of cells that did not increase over time. Only single infected cells were detected, and there was no development of plaques after 96 h (Figure 3a). In contrast, transfection of RK13 cells with DNA of parental EHV-1 typically resulted in extensive plaque formation after only 24 h (Figure 3b). Next, DNA of EHV-1ΔgH was transfected into RK13/gH1 as described above. Here, complete viral replication was evident and plaques similar in morphology to those induced by parental virus developed after 48 h (Figure 3c), although viral growth was not as robust as was the case for parental EHV-1. Finally, EHV-1ΔgH virus grown on RK13/gH1 cells was collected and used to infect naïve RK13 cells. Again, viral infection remained restricted to single cells (Figure 3d). In conclusion, our results showed that gH is essential for EHV-1 replication in vitro.

### Type-specific gH has no effect on host range of EHV-1 or EHV-4 in vitro

**Figure 4:**
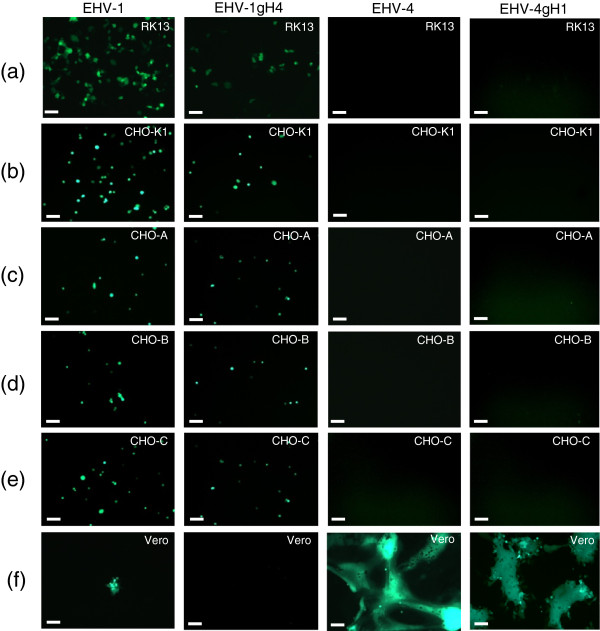
**The role of gH in EHV-1 and EHV-4 cellular tropism.** RK13 (**a**), CHO-K1 (**b**), CHO-A (**c**), CHO-B (**d**), CHO-C (**e**), or Vero (**f**) cells were infected at an MOI of 0.5 with the engineered virus recombinants, all of which express EGFP. At 48 h pi, cells were inspected with a fluorescent microscope (Zeiss Axiovert) and images were taken with a CCD camera (Zeiss Axiocam). The bar represents 100 μm.

**Figure 5:**
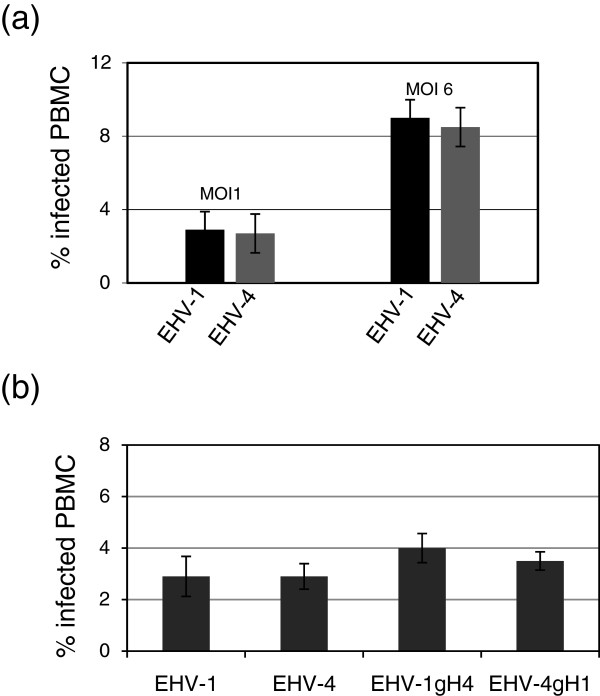
**Infection of equine PBMC with recombinant viruses.** PBMC were incubated with EHV-1 or EHV-4 at an MOI of 1 or 6 (**a**) for 1 h at 37°C. After 48 h, the percentage of infected cells was determined by flow cytometry. In another experiment, PBMC were infected with the engineered mutant viruses (**b**). The data represent the mean ± SD of at least three independent experiments.

EHV-1 can be readily propagated in many cell lines including primary equine cells or cell lines derived from other species [8]. In contrast, EHV-4 appears to be restricted mainly to cells derived from horses and replicates poorly in very few other cell lines such as Vero cells [38]. To test the hypothesis that gH might be a determinant of EHV-1 and EHV-4 host range, gH mutant viruses were used to examine the ability of the viruses to infect different cell lines in vitro. Our results showed that gH has no role in determining the host range of either EHV-1or EHV-4. EHV-1 parental and mutant viruses were still able to infect all cell lines that are permissive for EHV-1 and resistant to EHV-4, such as RK13 and CHO-K1 (Figure 4). However, viral spread was clearly affected by gH on RK13 cells, as was the case on NBL-6 cells (Figure 5a). Infection of Vero cells, which are permissive for EHV-4 but not EHV-1, showed that EHV-4 and EHV-4gH1 can infect these cells efficiently, while EHV-1 and EHV-1gH4 only poorly infected these cells (Figure 4). Finally, we found that HeLa, MDBK, PBMC, EC and 293 cells can be infected with parental and mutant viruses with similar efficiencies and that viral infection was independent of gH1 or gH4, respectively (data not shown).

### Integrins are not involved in EHV-1 or EHV-4 entry into PBMC or equine fibroblast cells

**Figure 6:**
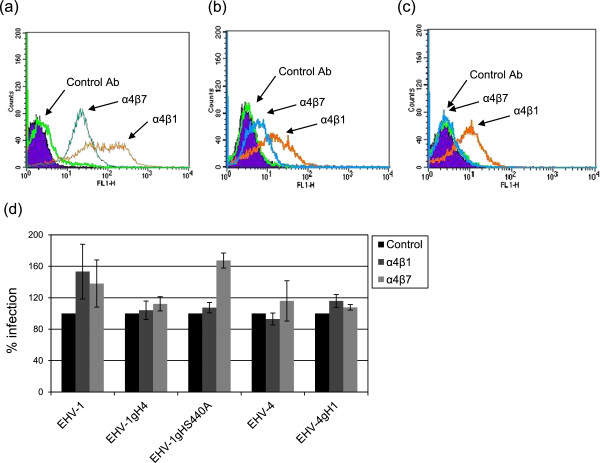
**Integrins have no role in EHV-1 or EHV-4 entry into equine cells.** PBMC (**a**) NBL-6 (**b**) or FHK (**c**) cells were incubated with anti-α4β7 MAb DATK32 or α4β1 MAb P4C2 for 1 h at RT, followed by incubation with Alexa fluor 488-labeled goat anti-mouse IgG for 1 h at RT. As controls, cells were incubated with irrelevant MAbs of the same IgG isotype. Integrin expression was determined by flow cytometry. (**d**) PBMC were preincubated with either α4β7 MAb DATK32 or α4β1 MAb P4C2 for 1 h at 4°C, followed by infection with recombinant viruses at an MOI of 5 for 2 h at 37°C. At 48 h pi, cells were washed and the percentage of infected cells was determined by flow cytometry. The rate of infection of parental viruses was set to 100%. All data represent the mean ± SD of three independent experiments.

PBMC are highly relevant to EHV-1 pathogenesis as outlined above, whereas EHV-4 infection of leukocytes is reported to be a rare event. It was postulated that the SDI integrin motif, present in EHV-1 gH but not EHV-4 gH, might be an important determinant for efficient infection of PBMC in vitro [6]. In order to address the hypothesis that gH1 and/or gH4 can affect viral infection of PBMC, we first infected PBMC with EHV-1 and EHV-4 strains and analyzed the percentages of infected cells by flow cytometry. Surprisingly, the percentage of infected PBMC after inoculation with EHV-1 or EHV-4 was nearly identical, ranging from approximately 3% (MOI = 1) to 9% (MOI = 6) (Figure 5a). In a second round of experiments, we included all of our mutants in the PBMC infection experiments. As with our previous results, the rate of infection was similar for all viruses used (Figure 5b). Furthermore, we used anti-integrin α4β1 (P4C2) or α4β7 (DATK32) MAb to investigate the role of integrins during entry into PBMC. First, the proper expression of α4β1 or α4β7 on PBMC using the function-blocking MAb P4C2 or DATK32 was confirmed (Figure 6a). Both α4β1 and α4β7 integrins were highly expressed on PBMCs, with 77.5% and 54.8%, respectively, of cells reactive with the antibodies. α4β1 integrins were also expressed on NBL-6 (40%) and FHK (30%) cells (Figure 6b and c), although there was no significant expression of α4β1 in either Vero or CHO-K1 cells (data not shown). The expression of the α4β7 integrin was minimal in NBL-6, FHK (Figure 6b and c), Vero or CHO-K1 cells (data not shown), and the mean fluorescence intensities not significantly different from that observed with mouse IgG control antibodies. Pre-incubation of PBMC with either α4β1 or α4β7 integrin-blocking antibodies did not lower the rate of viral infection in PBMCs for any of the parental or mutant viruses tested. Controls without antibodies for each virus were normalized to 100% and the percentage of infection after the use of antibodies was compared to this value. After using the α4β1 blocking antibody, infection rates of EHV-1 and related mutants were actually increased compared to controls without antibodies (Figure 6d), but this increase did not reach statistical significance for any of the viruses. In the case of EHV-4 there was a slight decrease in the percentage of infected cells, which was not significantly different from the average percentage of infection of the untreated virus. After treating cells with the α4β7 antibody, all viruses showed no significantly increased infection rates of PBMCs compared to untreated controls. Therefore, the integrin-blocking antibody does not have any significant adverse effect either on viral entry or on viral infection when added to cells prior to infection.

To further elucidate the role of integrins in virus entry, we either blocked α4β1 integrins on the surface of NBL-6 and FHK cells or incubated the viruses with soluble α4β1 integrin (15 μg/mL, R&D Systems, Minneapolis, USA) [39] before infection. Again, this treatment had no significant effect on the entry of parental or mutant viruses (data not shown). We concluded from our data that PBMC can be infected with similar efficiencies by both EHV-1 and EHV-4 in vitro. Furthermore, integrins may serve as a receptor and/or co-receptor for viral entry and their blockade may not have a measurable effect on virus infection, especially, if alternative receptors exist.

## Discussion

Alphaherpesviruses are successful at spreading directly from cell-to-cell in epithelial cells and neurons, thus avoiding the extracellular space and exposure to host defenses such as neutralizing antibodies. It is clear that the gE/gI heterodimer plays a major role in cell-to-cell spread, but gH also facilitates the movement of virus from infected to uninfected cells [40-42]. However, despite the fact that gH is involved in the process of cell-to-cell spread in a number of alphaherpesviruses [43], the actual role of gH in membrane fusion processes has yet to be elucidated. Here we show that EHV-1ΔgH replicates poorly in RK13 cells and does not form plaques, demonstrating the essentiality of EHV-1 gH in viral infection. The fact that the number of infected cells does not increase over time suggests that EHV-1ΔgH is not able to either enter cells or spread from an infected cell to adjacent uninfected cells [44]. By contrast, parental EHV-1 efficiently replicates in RK13 cells, producing a large number of plaques. The gH deletion virus was only able to produce plaques when grown in an RK13 cell line that was created to stably express gH1. A similar result was observed in the case of PRV and bovine herpesvirus-1 (BoHV-1) deleted in gH, where mutant viruses were unable to form plaques in non-complementing cells [42,45]. Furthermore, recombinant EHV-1 in which gH1 was replaced with gH4 produced significantly smaller plaques when compared to parental virus, whereas the insertion of gH1 into the EHV-4 genome led to increased plaque sizes. As seen above, the absence of gH in EHV-1 led to the complete abrogation of plaque formation; therefore, it is reasonable to assume that when gH1 is replaced with gH4, the mutant virus is less capable of engaging in cell-to-cell spread.

In a recent study conducted in our lab it was shown that, as is the case for other alphaherpesviruses, efficient EHV-1 and EHV-4 entry is mainly dependent on gD, which can bind to several cell surface receptors and determines the host range of both EHV-1 and EHV-4 [9]. In the study presented here, we found that, in all cells tested, the exchange of gH did not have an effect on the entry of the viruses into cells, thus indicating that gH is probably not important in determining cellular host range or binding to cellular receptors.

It has been shown that HSV-1 gB and gH/gL are involved in nuclear egress and fusion between the virion envelope and the outer nuclear membrane resulting in de-envelopment of primary enveloped viruses [46]. We, therefore, considered it likely that EHV-1 gH plays a role in viral egress, more specifically in the first envelopment step at nuclear membranes, secondary envelopment at cytoplasmic membranes, or the release of the virion into the extracellular space [47]. The fact that the exchange of gH had such a marked effect on virus cell-to-cell spread may indicate that there is little room to compensate for the loss of authentic gH in EHV-1 and/or that gH1 is much more efficient with respect to facilitating fusion.

Another point of consideration is the covalently linked heterodimer formed between gH and gL. The crystal structure of EHV gH has not been determined yet. However, on the basis of overall similarity, one may derive some information from other herpesviruses particularly HSV-2, PRV, and EBV. Biochemical data corroborated by crystal structure analyses revealed that it is the gL subunit and the N-terminal residues of gH that form the heterodimeric complex, which is essential for efficient membrane fusion and viral entry [19-22]. Furthermore, it has been established that gL is required for proper folding, intracellular transport, and full functionality of gH. The accumulated data and knowledge of the gH-gL interaction that make it difficult to evaluate the role of one of these glycoproteins individually as one has to assume that they will not act independently of each other but have to be viewed as a functional unit [48-51]. The N-terminal region of EHV-1 and EHV-4 gH share approximately 82% amino acid identity (Figure 1b). Perhaps, authentic gL could compensate for the phenotypic changes induced by the exchange of the respective gH’s. It will, therefore, be interesting to examine whether a double mutant, i.e. EHV-1 harboring gH4-gL4 and vice versa, would lead to any improvement or possibly even further impairment of the viruses’ ability to effectively replicate.

The amino acid motif LDV is the primary binding site for α4β1 integrins in fibronectin, and the related motifs LDI or SDI have been shown to bind α4 integrins with similar affinity and avidity [27-29]. To determine the role of integrins in viral entry into PBMCs, cells were incubated with antibodies directed against α4β1 or α4β7, and then infected with parental and mutant viruses. In PBMCs, none of the viruses were significantly affected by the presence of integrin-blocking antibodies. In most cases, viral entry into the cells was actually increased compared to cells not treated with antibodies. The same result was also obtained after blocking α4β1 integrin on the surface of equine fibroblasts. Furthermore, incubating either EHV-1 or EHV-4 with soluble α4β1 integrin before infection did not affect the entry. One may argue that integrin antibodies or soluble integrins were unable to block or mask either the integrin receptors on cell surface or the integrin motif present in gH. However, our results showed that both blocking integrin receptors and masking the integrin motif directed the virus to a different entry pathway, indicating reactivity and functional consequences of the antibodies and the soluble integrins (Azab and Osterrieder, unpublished data). The results of two recent reports support the findings of our experiments [9,52]. In these studies, it was shown that the presence of αVβ3 or αVβ5 integrins is not a requirement for either gD or gH-gL binding to different cells.

In summary, we conclude from our experiments that gH or the gH/gL complex is essential for the formation of plaques by EHV-1 and that the exchange of gH’s between EHV-1 and EHV-4 has a measurable impact on viral growth and plaque sizes. It remains unclear what this specific role of gH1 and gH4 in cell-to-cell spread entails, but similar results with related viruses suggest that the functions of gH and that of the heterodimeric gH-gL complex are at least partly conserved in the alphaherpesvirus subfamily. Lastly, our study has proven that integrins seem not to be essential for entry of EHV-1 or EHV-4 in any of the cell types analyzed.

## Competing interests

The authors declare that they have no competing interests.

## Authors’ contributions

WA and LZ have contributed equally to this work by designing and carrying out all the experiments, analyzing and interpreting the data. NO contributed to the drafting of the manuscript, revising it critically, and gave final approval of the version to be published. All authors read and approved the final manuscript.
